# The impacts of androgen receptor on treatment response and survival in triple-negative breast cancer treated with neoadjuvant chemotherapy: a single-center retrospective study

**DOI:** 10.3389/fonc.2026.1756969

**Published:** 2026-02-19

**Authors:** Seda Karaçam, Mehmet Furkan Sağdıç, Zarife Melda Bulut, Suat Kutun, Cihangir Özaslan

**Affiliations:** 1 Department of General Surgery, Sincan Research and Training Hospital, Ankara, Türkiye; 2 Department of Surgical Oncology, Ankara Etlik City Hospital, Ankara, Türkiye; 3 Department of Pathology, Ankara Oncology Training and Research Hospital, Ankara, Türkiye; 4 Department of Surgical Oncology, Gulhane Medical Faculty, Ankara Oncology Training and Research Hospital, Ankara, Türkiye

**Keywords:** androgen receptor, axillary lymph node metastasis, neoadjuvant chemotherapy, pathological response, triple negative breast cancer

## Abstract

**Background:**

Triple-negative breast cancer (TNBC) is an aggressive subtype characterized by the absence of estrogen receptor (ER), progesterone receptor (PR), and HER2 expression, resulting in limited treatment options. The present study explored the association between androgen receptor (AR) expression and clinicopathological features, as well as its impact on response to neoadjuvant chemotherapy (NACT) and its prognostic and predictive value in patients with TNBC.

**Method:**

In this single-center, retrospective study, we considered the data from 81 TNBC patients undergoing post-NACT surgery between January 1, 2017, and January 1, 2023, at the Ankara Oncology Health Application and Research Center of Health Sciences University (SUAM). Patients were grouped by their AR expression status and compared by clinicopathological features, treatment responses, and survival outcomes.

**Results:**

We detected AR positivity in 20 patients (26%) but found no significant association between AR expression and patients’ demographics. Yet, AR positivity was significantly associated with post-NACT axillary lymph node metastasis (p = 0.017), and the complete axillary response was significantly more prevalent in AR-negative patients (p = 0.002). Pre- and post-NACT Ki-67 values were significantly higher in the AR-negative group (p < 0.001 and p = 0.024, respectively). The findings showed no significant impact of AR status on disease-free survival (DFS) and overall survival (OS) (p = 0.132 and p = 0.079, respectively).

**Conclusion:**

Overall, we concluded that AR negativity was linked to increased proliferative activity and complete axillary response in TNBC patients. AR positivity, on the other hand, was associated with residual nodal disease. Ultimately, we could show no significant influence of AR expression on survival. Our findings suggest that AR may serve as a potential biomarker for predicting axillary response in TNBC.

## 1 Introduction

Triple-negative breast cancer (TNBC) is a relatively aggressive subtype, distinguished by the absence of estrogen receptor (ER), progesterone receptor (PR), and HER2 expression (1). This subtype accounts for about 10-15% of all breast cancer cases worldwide (2–4). In Turkey, data from the National Breast Cancer Registry Program indicate that TNBC constitutes approximately 8.1% of all breast cancer cases, demonstrating a comparable distribution to that reported globally (5). Due to the lack of receptors targeted by therapies such as anti-hormonal and targeted treatments, TNBC fails to respond to these modalities and tends to exhibit more aggressive behavior and poorer prognosis compared to other breast cancer subtypes (2, 3, 6, 7).

Androgen receptor (AR) expression in TNBC varies in the literature, ranging from 10% to 75% (8–11). It is involved in various processes such as proliferation, migration, invasion, and growth, both in normal breast tissue and breast tumors (12–14). The treatment challenges associated with TNBC have driven the search for new therapeutic targets and the development of alternative treatment strategies. Consequently, AR has become a focal point of interest and research. However, the prognostic impact of AR expression remains inconsistent in the literature. While some studies linked AR positivity with better overall survival (OS) and disease-free survival (DFS) compared to AR-negative cases (11, 15, 16), others found it to be associated with poorer OS and DFS (17, 18), and some studies reported no significant impact of AR positivity on OS and DFS (19, 20).

Ultimately, the present study explored the association between AR expression and clinicopathological features, as well as its impact on response to neoadjuvant chemotherapy (NACT) and its prognostic and predictive value in patients with TNBC.

This study focuses on a group of TNBC patients who underwent neoadjuvant chemotherapy—a subgroup that remains underrepresented in the literature. By exploring AR expression in relation to both axillary response and long-term outcomes, we hope to provide real-world insights that may support biomarker-guided treatment strategies in TNBC.

## 2 Materials and methods

In this single-center, retrospective study, we considered the data from 81 TNBC patients undergoing post-NACT surgery between January 1, 2017, and January 1, 2023, at the Ankara Oncology Health Application and Research Center of Health Sciences University (SUAM).

We performed *a priori* power analysis using the effect size (0.52) reported in the study by Mohammed et al., titled “Neoadjuvant Chemotherapy in Triple Negative Breast Cancer: Correlation between Androgen Receptor Expression and Pathological Response (21).” The sample size required for the Chi-square test (Goodness-of-fit: Contingency tables) was calculated using the G*Power software (G*Power 3.1 for Macintosh; Heinrich Heine University, Düsseldorf, Germany). Based on the analysis, with a Type I error rate of 0.05 and a beta value of 0.99, we determined the minimum required sample size for the study to be 68 (19). All methods were carried out in accordance with relevant guidelines and regulations, and with the Declaration of Helsinki. The study protocol was approved by the Ethics Committee of the University of Health Sciences, Ankara Oncology Training and Research Hospital (Approval No: 2021-06/1215). Written informed consent was obtained from all participants included in the study. In addition, approval for the procurement of test kits was granted by the Scientific Research Projects Coordination Unit of Health Sciences University, and the required funding was secured (Project No.: 2021/153).

TNBC patients who underwent post-NACT surgery and had AR expression assessed were included in the study. However, we excluded those who were metastatic at diagnosis and post-NACT patients with pathological complete response (pCR) due to unknown AR status.

All patients received anthracycline- and taxane-based neoadjuvant chemotherapy regimen (AC-T). None received immune checkpoint inhibitors or targeted therapies, as these were not yet in routine clinical use for TNBC in our center during the study period (2017–2023). Because of the regimen homogeneity, subgroup analysis by treatment protocol was not performed.

We collected the following data through the hospital database: patients’ demographic characteristics, menopausal status, diagnostic and post-NACT tumor size and lymph node status, Ki-67 proliferation index as determined by immunohistochemical (IHC) staining, tumor grade, and NACT response based on postoperative pathology reports, the total number of lymph nodes and positive nodes and nodal ratios for those undergoing axillary dissection, genetic test results (if accessible), and follow-up data including local recurrence, distant metastasis, and survival outcomes.

Total follow-up time was defined as the duration from the date of diagnosis to the date of last follow-up. DFS was calculated from the date of surgery to the first detection of either local recurrence or distant metastasis, if applicable. OS was defined as the time from diagnosis to death, or to the date of inclusion in the study for surviving patients.

Patients’ clinical and anatomical staging at diagnosis, as well as pathological and prognostic staging after NACT and surgery, were performed according to the current AJCC TNM staging system (22). We grouped patients by their clinical and pathological stages. While grouping patients into two (stage 1–2 and stage 3 patients) by clinical staging, we categorized them into two (stage 0–1 and stage 2–3–4 patients). All recorded parameters were compared by AR status.

### 2.1 Pathological evaluation

Formalin-fixed, paraffin-embedded tissue blocks obtained from pre-treatment core needle biopsies and post-neoadjuvant chemotherapy (NACT) surgical specimens containing residual tumor were retrieved from the institutional pathology archives. Sections (4 μm) with adequate tumor cellularity were prepared for immunohistochemical analysis.

Immunohistochemical staining for estrogen receptor (ER), progesterone receptor (PR), human epidermal growth factor receptor 2 (HER2), and androgen receptor (AR) was performed using the BenchMark ULTRA^®^ automated staining system (Roche Diagnostics, Basel, Switzerland), in accordance with the manufacturer’s instructions. Primary antibodies included anti-ER (SP1), anti-PR (1E2), anti-HER2/neu (4B5), and anti-AR (SP107). Detection was achieved using the UltraView Universal DAB Detection Kit^®^, followed by hematoxylin counterstaining.

Triple-negative breast cancer was defined according to current American Society of Clinical Oncology/College of American Pathologists (ASCO/CAP) guidelines (23, 24). Tumors were considered ER- and PR-negative if less than 1% of tumor cells demonstrated nuclear staining. HER2 status was evaluated based on membrane staining intensity and completeness; cases scored as 2+ underwent further assessment using silver *in situ* hybridization (SISH).

All patients included in the study underwent surgery following completion of neoadjuvant chemotherapy (NACT). Therefore, the only pre-treatment tissue samples available were tru-cut core needle biopsies, which were often limited in size and quantity. Due to the small volume and fragmented nature of these specimens, it was not technically feasible to perform AR immunohistochemistry (IHC) on all of them retrospectively. Furthermore, for patients achieving pCR after NACT, AR could only be assessed in biopsy specimens. When sufficient pre-NACT biopsy material was available and residual tumor tissue remained after NACT, AR immunostaining was performed on both core biopsy samples and post-NACT surgical specimens. However, no formal comparative analysis was planned or performed, as evaluation of temporal changes in AR expression was not within the primary objective of this study.

**Figure 1:**
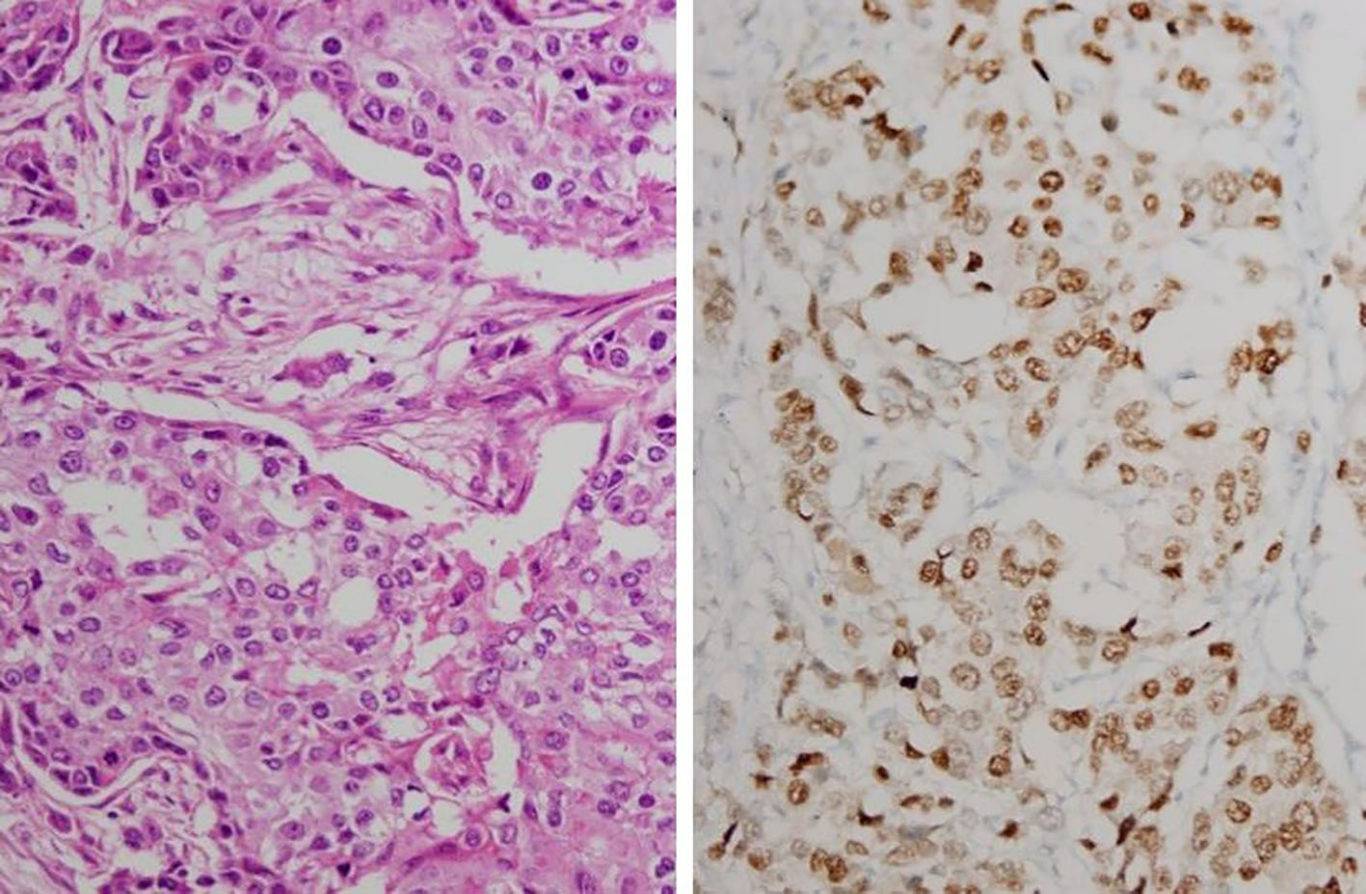
Invasive breast carcinoma: IHC staining. H&E staining (left) and 60% staining pattern with AR (right) (x400).

Since it is not routine to evaluate AR in current clinical practice for breast cancer, the ASCO/CAP guidelines (23) established for ER and PR evaluation was adapted for AR assessment in this study as commonly practiced in the literature. The AR cut-off was defined as <1% nuclear staining, consistent with the guidelines’ principles used for ER and PR evaluation. For each AR-stained slide, the corresponding hematoxylin and eosin-stained slide of the same tissue was simultaneously reviewed to define the tumor region. Within this designated area, nuclear AR staining was evaluated jointly by an experienced pathologist (Z.M.B.) and a surgeon (principal researcher – S.K.) under a light microscope. The percentage of AR-positive tumor cells was calculated by dividing the number of stained tumor cell nuclei by the total number of tumor cells observed in the tumor area (**Figure 1**).

### 2.2 Statistical analysis

Descriptives are presented as number (n), percentage (%), mean (M), standard deviation (SD), and median (minimum-maximum). As a univariate analysis of the data, we utilized chi-square and Fisher’s exact tests. Survival outcomes were assessed using the Kaplan-Meier method, while survival differences were compared with the Log-rank test. We report results within a 95% confidence interval (CI), with statistical significance set at p < 0.05. We performed all statistical analyses using SPSS v.25 (SPSS Inc.; Chicago, IL, USA).

## 3 Results

We retrospectively reviewed the records of patients who were diagnosed with triple-negative breast cancer (TNBC) between January 1, 2017, and January 1, 2023, received NACT, and subsequently underwent surgery at SUAM. We analyzed the data from 81 patients satisfying our inclusion criteria by their AR status, as determined either from trucut biopsy or postoperative surgical specimens. While categorical variables are summarized in **Table 1**, continuous variables are presented in **Table 2**.

**Table 1:** Patients’ clinical and pathological features.

Clinicopathological variables	AR-negative n=61 (%)	AR-positive n=20 (%)	p-value
Age (years) ≤50 >50	29 (47.5) 32 (52.5)	10 (50) 10 (50)	0.849
Menopausal Status Premenopausal Postmenopausal	30 (49.2) 31 (50.8)	8 (40) 12 (60)	0.475
Surgery Mastectomy BCS	43 (70.5) 18 (29.5)	17 (85) 3 (15)	0.414
SLNB Yes No (Dissection)	38 (62.3) 23 (37.7)	6 (30) 14 (70)	0.009*
Axillary Dissection Yes No	29 (47.5) 32 (52.5)	16 (80) 4 (20)	0.011*
SLNB -/+ Negative Positive N/A	32 (53) 6 (10) 23 (37)	4 (20) 2 (10) 14 (70)	0.040*
Pre-NACT Histopathology NST Metaplastic Others	56 (91.8) 3 (4.9) 2 (3.3)	19 (95) 1 (5) 0	0.723
Post-Surgery Histopathology NST ILC Metaplastic Others No residual tumor	34 (55.7) 0 7 (11.5) 3 (4.9) 17 (27.9)	16 (80) 1 (5) 1 (5) 0 2 (10)	0.176
Pre-NACT T-stage T1 T2 T3	10 (16.4) 41 (67.2) 10 (16.4)	1 (5) 12 (60) 7 (35)	0.252
Post-Surgery T-stage T1 T2 T3 pCR	14 (23) 18 (29.5) 12 (19.7) 17 (27.8)	3 (15) 7 (35) 8 (40) 2 (10)	0.325
Pathological Response Complete Partial None	17 (27.9) 22 (36.1) 21 (34.4)	2 (10) 9 (45) 9 (45) -	0.248
Pathological Response Complete None	16 (26) 45 (73)	2 (10) 18 (90)	0.130
Pre-NACT Nodal Status Positive Negative	45 (73.8) 16 (26.2)	15 (75) 5 (25)	0.110
Post-NACT Nodal Status in Patients with Axillary Dissection Positive Negative No dissection	18 (29.5) 15 (24.6) 28 (45.9)	13 (65) 1 (5) 6 (30)	0.017*
Post-NACT Nodal Status in Node Positive Patients Non-pCR pCR Pre-NACT Node Negative	18 (29.5) 27 (44.3) 16 (26.2)	13 (65) 2 (10) 5 (25)	0.002*
Nodal Response Complete Axillary Response No axillary response Pre-NACT Node Negative	28 (50) 17 (28.3) 16 (26.7)	2 (10.5) 14 (68.4) 4 (21.1)	0.002*
Pre-NACT Ki-67 ≤30 >30 missing	2 (3.3) 57 (93.4) 2 (3.3)	5 (25) 12 (60) 3 (15)	0.008*
Post-NACT Ki-67 ≤30 >30 No Residual Tumor	4 (6.5) 40 (65.6) 17 (27.9)	2 (10) 15 (75) 3 (15)	0.753
Pre-NATC Grade Grade 1-2 Grade 3	10 (16.4) 51 (83.6)	6 (30) 14 (70)	0.206
Post-Surgery Grade Grade 1-2 Grade 3 No Residual Tumor	10 (16.4) 34 (55.7) 17 (27.9)	4 (20) 14 (70) 2 (10)	0.930
Pre-NACT Stage 1-2 3	47 (77) 14 (23)	13 (65) 7 (35)	0.378

*NAC: Neoadjuvant Chemotherapy.

**BCS: Breast Conserving Surgery.

***SLNB: Sentinel Lymph Node Biopsy.

Bold values indicate statistically significant results (p < 0.05).

**Table 2:** Patients’ clinical and pathological features.

Clinicopathological variables	AR-negative n=61 (%)	AR-positive n=20 (%)	p-value
Age (years) Mean Median	50.4 ± 10.9 50 (28-74)	52.6 ± 12.5 49 (38-80)	0.457
Pre-NACT Radiological Tumor Size (mm) Mean Median	36.8 ± 20.9 33 (10-100)	36.9 ± 17.4 30 (16-76)	0.843
Post-NACT Tumor Size (mm) Mean Median	38.47 ± 37.7 23.5 (0-170)	51.33 ± 45 40.5 (3-175)	0.136
Axillary dissection - total lymph node count Mean Median	16.48 ± 9.52 6 (1-34)	17.5 ± 11 11 (1-39)	0.745
Axillary dissection – positive lymph node count Mean Median	1.05 ± 2.43 0 (1-34)	5.70 ± 8.71 2.5 (1-39)	<0.001*

Bold values indicate statistically significant results (p < 0.05).

At the time of diagnosis, the mean age was 50.4 ± 10.9 years in the AR-negative group [median: 50 (28–74)] and 52.6 ± 12.5 years in the AR-positive group [median: 49 (38–80)] (p = 0.457). Of patients, 75 (92.5%) were initially diagnosed with invasive breast carcinoma, while 4 (5%) had metaplastic carcinoma, and 2 (2.5%) presented with rare subtypes (adenoid cystic carcinoma and neuroendocrine carcinoma) (p = 0.723).

Sixty-two patients had residual tumors in the specimen following surgery: 50 (80.6%) were diagnosed with invasive breast carcinoma, 8 (12.9%) with metaplastic carcinoma, 3 (4.8%) with rare subtypes (encapsulated papillary carcinoma, adenoid cystic carcinoma, and neuroendocrine carcinoma), and 1 (1.6%) with invasive lobular carcinoma (p = 0.176).

Radiological assessment of pre-NACT tumor size demonstrated similar long-axis measurements in both groups: 36.8 ± 20.9 mm in the AR-negative group and 36.9 ± 17.4 mm in the AR-positive group (p = 0.843). Among patients with residual tumors after surgery, the mean tumor size based on pathological examination of the surgical specimen was 38.47 ± 37.7 mm for AR-negative and 51.33 ± 45 mm for AR-positive patients (p = 0.136).

When assessing pathologic response to NACT, 19 out of 80 patients (23.75%) achieved a complete response, 31 (38.75%) had a partial response, and 30 (37.5%) showed no response. Pathological complete response was observed in 17 of 60 patients (28.33%) in the AR-negative group and in 2 of 20 patients (10%) in the AR-positive group. There was no statistically significant association between AR status and pathologic response to NACT (p = 0.248).

Clinically detected lymph node (LN) involvement was observed before NACT in 60 patients (74%), of whom 45 (75%) were AR-negative and 15 (25%) were AR-positive. Among the 21 patients (26%) with no clinical pre-NACT LN involvement, 16 (76.2%) were AR-negative, and 5 (23.8%) were AR-positive (p = 0.110).

Sixty patients (74%) underwent a mastectomy, while 21 (26%) received breast-conserving surgery (BCS). Sentinel lymph node biopsy (SLNB) was performed in 44 patients (54%), whereas 37 patients (46%) underwent axillary dissection due to confirmed nodal involvement. Among the 44 patients undergoing SLNB, 36 (80%) had negative results, and 9 (20%) required subsequent axillary dissection due to SLNB positivity.

While 61 patients (75%) were node-positive, 20 patients (25%) were node-negative prior to NACT. Among the AR-negative patients with pre-NACT nodal involvement, 28 (50%) achieved a complete axillary response following treatment, whereas 17 (28.3%) did not. In contrast, only 2 (10%) AR-positive patients demonstrated a complete axillary response, but 14 (68.4%) did not. These findings suggest a significantly lower rate of complete axillary response among AR-positive patients (p = 0.002).

In axillary dissection patients, the mean number of excised lymph nodes was 16.48 ± 9.52 in the AR-negative group (n = 33, 70.2%) and 17.5 ± 11 in the AR-positive group (n = 14, 29.8%) (p = 0.745). The mean count of pathologically confirmed metastatic lymph nodes in the AR-negative group (1.05 ± 2.43) was significantly lower than that in the AR-positive group (5.70 ± 8.71; p < 0.001). Among those with clinically confirmed pre-NACT axillary involvement, 29 (48.3%) achieved a complete axillary response, whereas 31 (51.7%) still had residual nodal disease following treatment.

We found that while 27 AR-negative patients achieved a complete axillary response, this was the case for only 2 AR-positive patients. This finding suggests a significant association between axillary response to NACT and AR status, in favor of AR-negativity (p = 0.002).

The mean pre-NACT Ki67 index was 71.44% in the AR-negative group (n = 59) and 45.63% in the AR-positive group (n = 19), revealing a significantly higher proliferative index in the AR-negative group (p < 0.001). Similarly, we found a significant association between AR-negativity and Ki67 values ≥ 30 (p = 0.008).

Post-surgical Ki67 values were also assessed among patients with residual tumors. Accordingly, it was found to be 67.3% in the AR-negative group (n = 44) and 51.7% in the AR-positive group (n = 17), with the difference being significant (p = 0.024). Nevertheless, unlike the pre-NACT findings, there was no significant association between AR status and post-surgical Ki67 categories (≥30 vs. <30%; p = 0.753).

Prior to NAC, 16.4% of AR-negative patients (n = 10/61) had Grade 1–2 tumors, and 83.6% (n = 51) had Grade 3 tumors. These rates were calculated to be 30% (n = 6) and 70% (n = 14), respectively, among AR-positive patients. The pre-NACT tumor grade did not significantly differ between the groups (p = 0.206).

We discovered a similar pattern in post-surgery patients with residual tumors: 22.7% of the AR-negative group (n = 10) had Grade 1–2 tumors, and 77.3% (n = 34) had Grade 3 tumors; 22.2% of the AR-positive group (n = 4) had Grade 1–2 tumors, and 77.8% (n = 14) had Grade 3 tumors. As with pre-NACT findings, we could not conclude a significant difference between postoperative tumor grade by AR status (p = 0.930).

**Figure 2:**
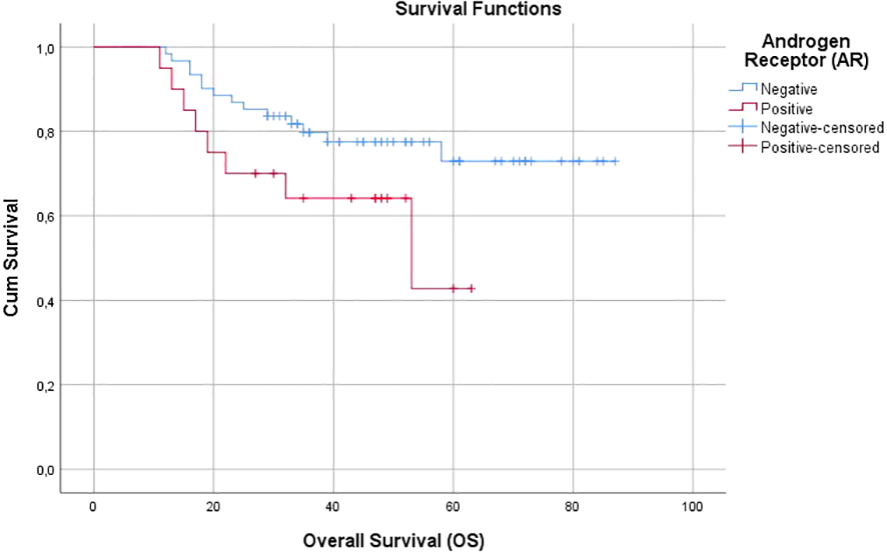
Overall Survival (OS).

**Figure 3:**
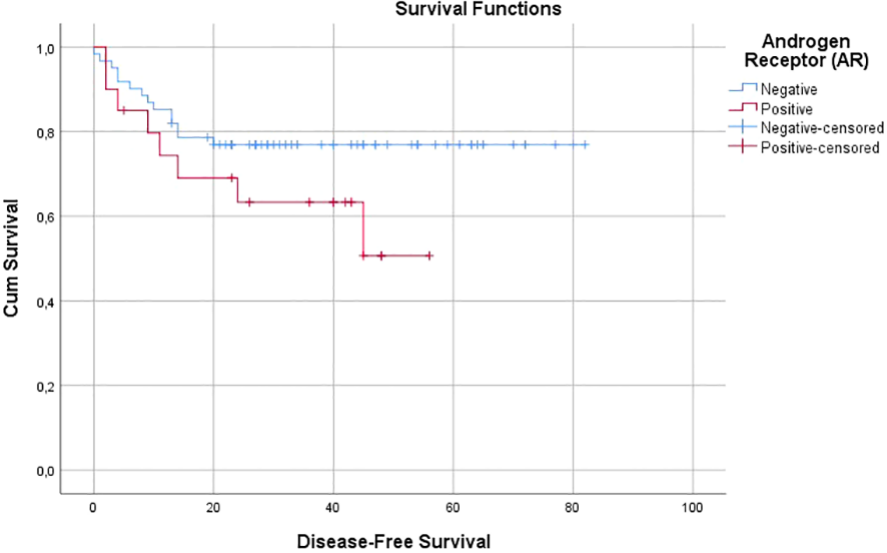
Disease-Free Survival (DFS).

The mean OS was found to be 45 months in the AR-positive group and 71 months in the AR-negative group (p = 0.079; **Figure 2**). Similarly, the mean DFS was 37 months in the AR-positive group and 65 months in the AR-negative group (p = 0.132; **Figure 3**). The 5-year OS rate was 42.8% (95% CI: 35.5%–54.5%) in the AR-positive group, whereas it was 73% (95% CI: 64.2%–78.6%) in the AR-negative group.

Five patients (5%) were with local recurrence, with 4 of them (7.4%) in the AR-negative group and one (6.3%) in the AR-positive group (p = 1.00). Distant metastasis occurred in a total of 19 patients (23.4%). These patients were distributed as 24% (n = 13) in AR-negative cases and 38% (n = 6) in AR-positive cases (p = 0.343).

## 4 Discussion

Currently lacking an established targeted therapy beyond conventional chemotherapy, TNBC may be subdivided into distinct molecular subtypes to facilitate the development of more efficient and personalized treatment modalities. In this regard, it was previously hypothesized that AR, known to play a role in cell proliferation and tumor progression in breast cancer, may function similarly to ER in TNBC by inducing cellular proliferation through transcriptional activation (25–27). Grounded in this hypothesis, our study aimed to examine the prognostic significance of AR in TNBC by both patients’ demographic and clinicopathological characteristics, as well as their survival outcomes, which is believed to contribute to a growing body of literature hosting varying and sometimes contradictory findings regarding the role of AR in TNBC (7, 28–46).

AR positivity in TNBC varies widely across studies, ranging from 8.3% to 58.6% (9, 38, 42, 46). In our study, we identified AR positivity in 26% of patients. While some studies indicated that AR-positive patients tend to be older (38, 46), others found no correlation between AR status and age (29, 33, 47). Consistent with the latter, our findings did not demonstrate a significant association between AR expression and age. Similarly, although previous research reported higher AR expression in both postmenopausal (19, 48) and premenopausal patients (37), we found no significant relationship between menopausal status and AR expression in our cohort.

The rate of post-NACT pCR in TNBC ranges from 40% to 50% in the literature (49–51). In a study by Li et al., pCR rates were lower among AR-positive TNBC patients compared to their AR-negative counterparts (52). Similarly, Leone et al. found that AR-negative patients had higher pCR rates and Ki-67 levels (53), aligning with the findings of Masuda et al., who associated the LAR subtype with lower pCR rates (54). In contrast, while Dieci et al. identified a clear association between AR negativity and elevated Ki-67, they found no significant correlation between AR status and pCR (44). Sankhyadhar et al. reported a significant link between AR positivity and Ki-67 expression (55).

When it comes to our findings, the AR-negative group had significantly higher Ki-67 levels both before NACT (p < 0.001) and postoperatively (patients with residual tumors; p = 0.024). However, there was no significant association between AR status and pCR in the primary tumor (p = 0.130), which may be attributable to our relatively limited sample size. Although pCR encompasses response in both the primary tumor and axillary nodes, we analyzed these outcomes separately. Accordingly, AR status was not associated with pre-NACT axillary involvement, and postoperative axillary metastasis was significantly more frequent in AR-positive patients with initial axillary involvement (p = 0.017). Furthermore, among patients undergoing post-NACT axillary dissection, the number of metastatic lymph nodes identified in the surgical specimen was significantly higher in the AR-positive group (p < 0.001). Notably, patients who had clinical pre-NACT axillary involvement but achieved a complete axillary response postoperatively were predominantly AR-negative. Nevertheless, previous findings regarding the relationship between AR status and axillary metastasis remain inconsistent. Wang et al. reported a higher incidence of AR positivity in patients with axillary lymph node metastasis (19). The literature hosts similar observations, all indicating an increased likelihood of axillary lymph node metastasis among AR-positive patients (7, 30, 46). Conversely, Zaborowski and He et al. found axillary involvement to be more prevalent in AR-negative cases (56, 57). In addition, there are studies reporting no significant association between AR status and axillary metastasis (16, 37).

Previous research exploring the relationship between tumor grade and AR expression yielded conflicting results. While some studies associated AR expression with lower tumor grades (8, 29, 39, 44, 46), others reported no significant link between AR status and tumor grade (11, 38). In their meta-analysis, Wang et al. found AR positivity to be significantly associated with Grade 1 and 2 tumors (19). Overall, the majority of published data appear to support a link between AR expression and lower tumor grade. In our study, however, we did not conclude a significant association between AR status and tumor grade, either at diagnosis or following surgery after NACT in patients with residual tumors (p = 0.206).

The impact of AR expression on survival outcomes in TNBC patients remains inconclusive in the literature. In their meta-analysis, Wang et al. reviewed 13 studies with a total of 2,826 patients and found AR positivity to be significantly associated with longer DFS; however, this was not the case with OS (19). In another meta-analysis, Xu et al. reviewed 27 studies involving 4,914 patients, reported an overall AR expression rate of 27.96%, and found no significant relationship between AR status and DFS, OS, distant disease-free survival (DDFS), or recurrence-free survival (RFS) (20).

In a multicenter study including 1,407 patients from the US, UK, Norway, Ireland, Nigeria, and India–all evaluated with a uniform IHC protocol–AR positivity was associated with favorable prognosis in the US and Nigerian cohorts, unfavorable prognosis in the Norwegian, Irish, and Indian cohorts, and remained neutral in the UK cohort (42). Similarly, a recent meta-analysis with 15 cohort studies comprising 2,713 TNBC patients found no significant relationship between AR status and either OS or DFS (58). Moreover, Dubrava et al. and Di Leone et al. reported no significant correlation between AR expression and OS or DFS (38, 53). In line with these findings, our study did not identify a significant association between AR expression and OS, DFS, or local recurrence. These results suggest that population-specific factors may play a key role in disease prognosis. In addition, heterogeneity in IHC protocols across studies likely contributes to the inconsistency of the findings.

This study has several limitations. Due to its retrospective design and moderate sample size, AR expression was evaluated using a binary definition of positivity (≥1% nuclear staining), based on standard hormone receptor guidelines. Because AR assessment is not routinely standardized in clinical breast cancer practice and no universally accepted AR-specific scoring system exists, cut-off thresholds established by the ASCO/CAP guidelines for estrogen and progesterone receptor evaluation were adapted for AR assessment in this study, consistent with commonly adopted approaches in the literature. Although weak versus strong AR positivity may carry different biological and clinical implications, subgroup analyses based on staining intensity were not feasible in our cohort.

In addition, AR immunohistochemistry could not be performed in all pre-treatment biopsy samples due to limited or fragmented tru-cut material. In patients with sufficient tissue, AR expression was assessed in pre-treatment biopsies and/or post-treatment surgical specimens when residual tumor was present; however, a systematic paired analysis could not be conducted because of limited tissue availability and the absence of residual tumor in patients achieving pathological complete response. Furthermore, although tumor stage distributions differed numerically between AR-positive and AR-negative groups, stage-wise subgroup analyses were not performed due to the limited sample size and the risk of underpowered statistical comparisons.

Another limitation is that all patients received anthracycline- and/or taxane-based neoadjuvant chemotherapy. Therefore, we could not evaluate whether AR expression might show different associations with treatment response in other chemotherapy regimens or in patients receiving targeted therapies. Furthermore, although adjuvant capecitabine was administered to a considerable proportion of patients with residual disease in both AR-positive and AR-negative groups, its use was heterogeneous and not standardized according to a uniform protocol, precluding meaningful adjustment for adjuvant treatment effects in the analysis. In addition, molecular profiling using next-generation sequencing (NGS) could not be performed, both due to limited tissue availability and the lack of institutional resources for such analysis. Future studies with prospective design and molecular subtyping may provide better insights, particularly for identifying the luminal androgen receptor (LAR) subtype among TNBC patients.

Despite several limitations of our study, including its retrospective nature, relatively small sample size, and short follow-up period, we believe that our findings contribute to the existing literature and may provide useful guidance for future research. In patients with TNBC, AR expression might be a helpful biomarker to assess axillary nodal response and predict axillary metastasis following neoadjuvant chemotherapy. As there are still no established targeted therapies for TNBC, AR inhibition could be considered as a potential treatment option.

Although many studies have investigated the prognostic role of AR in triple-negative breast cancer, our study offers additional value by focusing on the association between AR expression and treatment response, as well as survival, specifically in patients receiving neoadjuvant chemotherapy. This group is clinically relevant but often underrepresented in studies. Moreover, the observed relationship between AR status, axillary response, and recurrence strengthens the clinical significance of our results. We also believe that real-world data from our single-center Turkish cohort may support the development of biomarker-based treatment approaches in TNBC.

## 5 Conclusions

To sum up, the results showed a significant relationship between AR negativity and higher Ki-67 levels in our cohort of TNBC patients undergoing post-NACT surgery. This was also the case between AR negativity and post-NACT complete axillary response. There was also a significant relationship between AR positivity and post-NACT residual axillary disease. Moreover, AR positivity was associated with a higher number of metastatic lymph nodes detected in axillary dissection specimens following NACT.

## Data Availability

The raw data supporting the conclusions of this article will be made available by the authors, without undue reservation.
